# Quality of life measures in older adults after traumatic brain injury: a systematic review

**DOI:** 10.1007/s11136-019-02297-4

**Published:** 2019-09-14

**Authors:** Cindy Hunt, Shatabdy Zahid, Naomi Ennis, Alicja Michalak, Cheryl Masanic, Chantal Vaidyanath, Shree Bhalerao, Michael D. Cusimano, Andrew Baker

**Affiliations:** 1 Head Injury Clinic, Trauma and Neurosurgery Program, St. Michael’s Hospital, Dalla Lana School of Public Health, University of Toronto, Toronto, ON Canada; 2 University of Toronto, Toronto, ON Canada; 3 Department of Psychology, Ryerson University, Toronto, ON Canada; 4 St. Michael’s Head Injury Clinic and UHN Toronto Rehabilitation Clinic Toronto, Toronto, ON Canada; 5 Department of Surgery, Division of Neurosurgery, Injury Prevention Research Office, Keenan Research Centre, St. Michael’s Hospital, University of Toronto, Toronto, ON Canada; 6 Departments of Anesthesia and Critical Care, Keenan Research Centre for Biomedical Science, St. Michael’s Hospital Toronto, University of Toronto, Toronto, ON Canada; 7 St. Michael’s Head Injury Clinic, Toronto, ON Canada

**Keywords:** Traumatic brain injury, Quality of life, Literature review, Older adult

## Abstract

**Background:**

On average older adults experiencing TBI are hospitalized four times as often, have longer hospital stays, and experience slower recovery trajectories and worse functional outcomes compared to younger populations with the same injury severity. A standard measure of Qol for older adults with TBI would facilitate accurate and reliable data across the individual patient care continuum and across clinical care settings, as well as support more rigorous research studies of metadata.

**Purpose:**

The aim of this systematic review was to investigate patient reported Qol measures in studies with older adults post TBI.

**Method:**

A systematic review was carried out focusing on the various tools to measure Qol in older adults, ≥ 65 years of age with a diagnosis of TBI. Data bases searched included Medline, Embase, PubMed, CINAHL, and PsychInfo from date of inception to September 25, 2017.

**Results:**

A total of 20 articles met the inclusion criteria. Nine different tools were identified.

**Conclusions:**

Findings based on the comparison of reliability and construct validity of the Qol measures reported in this review suggest that no single instrument is superior to all others for our study population. Future research in this field should include the enrollment of larger study samples of older adults. Without these future efforts, the ability to detect an optimal Qol measure will be hindered.

## Background

The rate of hospitalization among older adults (≥ 65 years of age) with traumatic brain injury (TBI) has increased by 24% over the past decade [1]. On average, older adults with TBI are hospitalized four times as often, have longer hospital stays [2], and experience slower recovery trajectories and worse functional outcomes compared to younger populations with the same injury severity after TBI [3]. The economic cost of TBI is expected to rise from $7.3 billion in 2011 to $8.2 billion (CAD) in 2021 [2]. Much of what has been studied about the personal impact of TBI on older adult quality of life (Qol)—both in acute and rehabilitative care—has been based on symptom reduction or information provided by family or clinician ratings. Only recently, has the importance of the patient’s perspective on Qol become a critical indicator following TBI [4, 5]. Qol is conceptualized as self-reported overall contentment across different areas of life, including physical well-being, social relationships, community activities and recreation, and personal fulfillment [6]. Reviews with a focus on childhood TBI highlight the challenges of drawing conclusions across studies when study measures differ [7–9]. Trauma in older adult patients has been insufficiently studied [10]. The lack of a standard Qol measure for older adults contributes to this knowledge deficit. Implementing a common measure of Qol can help improve the care received by the older adults and increase our understanding of their unique needs.

A standardized Qol measure would assess the effectiveness of interventions [5, 11]. A standardized Qol measurement for older adults post TBI could improve clinical practice, enhance health care delivery, inform health policy, and support allocation of health service funds [12]. Local-level [13] and international [14] TBI research has recognized the importance of using standardized measures to improve data quality and patient care. A standard QoL measure for older adults with TBI could facilitate accurate and reliable data within an individual patient care continuum and across clinical care settings, and support more rigorous research studies with metadata.

We conducted a systematic review with the aim to identify Qol measurement tools used for older adults post TBI, and examined the psychometric properties and feasibility of administration among older adults, to aid in the identification of a standardized Qol tool for this population.

## Methods

The systematic literature review followed the Preferred Reporting Items for Systemic Review and Meta-Analysis (PRISMA) statement [15]. The study protocol was documented in PROSPERO [16], Registration Number: CRD42018092730.

### Study eligibility criteria

The studies met the following inclusion criteria:

1. Study participants experienced TBI of any severity (mild, moderate, severe);
2. The study author explicitly referred to the tool as measuring Qol;
3. The Qol measure assessed more than one domain;
4. Evidence of at least 5 study participants that were ≥ 65 years at time of Qol assessment;
5. If a study included adults with conditions other than TBI, data on the participants with TBI must have been examined and reported separately from other group(s).

### Information sources

Search terms for the databases were first developed by the authors in consultation with an information specialist. Our population included various terms for older adults such as “aged,” “pensioner,” and “aged 65 years and above.” We included search terms for quality of life measures such as specifying tools, health indicators, and health-related quality of life (see Appendix). The computerized search strategy was peer-reviewed by a second information specialist in accordance with PRISMA guidelines. Once approved, the search was conducted in the following databases: Medline, Embase, PubMed, CINAHL, and PsychInfo from date of inception to September 25, 2017. The search was limited to the English language. The full electronic search strategy for one database (Medline) is presented in Appendix.

### Study selection

Three researchers (CH, NE, SZ) independently screened 3586 titles and abstracts against the inclusion criteria. We discussed our individual results, and disagreements were resolved through consensus. In total, 508 full texts were assessed for eligibility. After full-text review, we yielded 20 studies for quality assessment and data abstraction.

Two authors (CH, SZ) independently reviewed the included full-text studies and extracted information to a spreadsheet listing geographic location, level of care, study design, sample size, participant characteristics: sex, mean age of study participants, age range, frequency and proportion of total participants aged 65 and older, and TBI severity (mild, moderate, or severe). Data on Qol instruments was extracted from each study and recorded by name of Qol measure(s), type and number of dimensions, administrative time point(s), psychometric properties using the COSMIN guideline [17], and feasibility among older adults. Data were abstracted into the spreadsheet independently and disagreements were resolved through consensus.

### Quality assessment of individual studies

The Downs and Black checklist [18] with revisions [19–21] was used to evaluate methodological quality of each study that met the criteria for quality assessment. Revisions to the Downs and Black [18] checklist for non-intervention studies entailed removal of items 4, 8, 13, 14, 15, 19, 21–24 inclusive, because the items were not relevant to observational studies. Item number 5 was re-valued to have potential value of 2 points if the study included socioeconomic status as a confounder among others, 1 point if it mentioned confounders but not socioeconomic status, and 0 points if it did not mention any confounders. Socioeconomic status is an important confounder to consider as it affects accessibility to services among older adults [11]. Item 27 which addressed statistical power and scoring was simplified from a 5-point scale to a 0 or 1 point score. One point was awarded if the study power or sample size was reported and a score of 0 was awarded where no sample size, no power calculation, or no explanation as to whether the number of subjects was appropriate for the question being asked was reported [11]. Higher scores on the Downs and Black checklist indicated a greater degree of methodological quality. Given the revisions, each study could achieve a maximum score of 18 points. The exception to the revisions was a single randomized clinical trial study [22]. As such, all items on the Downs and Black checklist were included in the quality assessment, which had a total potential score of 28 points. Inter-rater reliability measured by Cohen’s Kappa = .84 between the two authors completing the quality assessment data [23] with disagreements resolved by discussion. Qol measures were considered if one additional publication tested the psychometrics of the specific Qol measure.

## Results

### Study selection

**Fig. 1:**
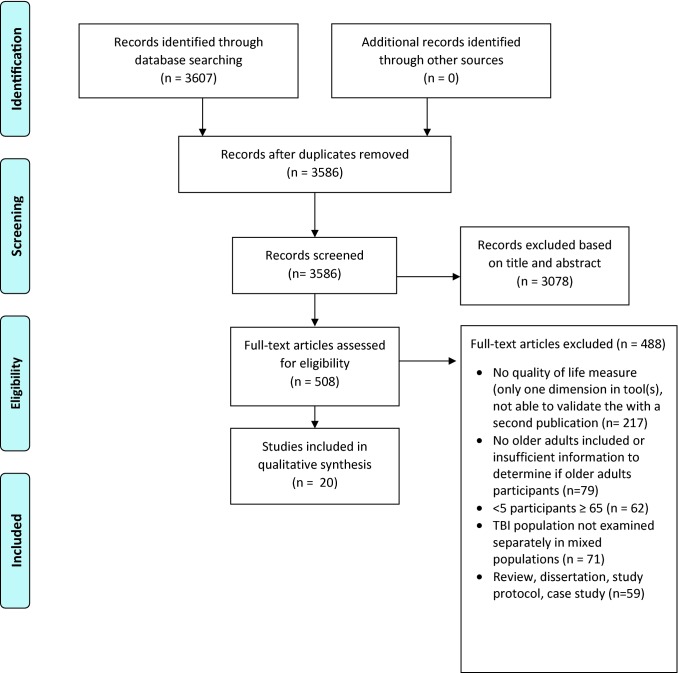
PRISMA Flowchart of studies

The search yielded 3607 articles, of these 21 were duplicates, and 3078 were excluded based on the titles and abstracts that failed to meet inclusion and exclusion criteria, thereby leaving 508 full-text articles to be assessed for eligibility. After assessing eligibility of full-text articles, we included 20 studies for quality assessment and data abstraction [22, 24–42]. The PRISMA flow diagram is shown in Fig. 1. Eight papers identified in the search included older adults according to the age range, but did not report the number of adults, ≥ 65 years. The authors were contacted by email in order to identify if the paper met our review inclusion criteria of 5 or more older adults. However, we did not receive a response and these studies were excluded.

### Study characteristics

#### Geographical location, level of care, and study design

The studies selected for review represented 12 different populations worldwide. These included United States [25, 29, 30, 34, 36, 39, 41], Canada [31, 38], England [27, 40], Spain [33], Brazil [42], France [24], Switzerland [28], Taiwan [32], Sweden [22], Germany [35], Netherlands [26], and China [26] (Table 1). Levels of care among the 20 studies included 10 community care environments [25, 29, 30, 35–37, 39–42], 8 hospital settings [22, 26–28, 31, 33, 34, 38], and 2 studies that recruited participants from both community and hospital [24, 32]. Half of the studies were cohort design [25–28, 31, 32, 36, 38, 39, 42], nine were cross sectional surveys [25, 29, 30, 33, 35, 37, 40, 41], and one was a randomized control trial [22]. Pickelsimer et al. [36] and Selassie et al. [37] used the same study sample, which was determined based on the review of the sample size, mean age of study participants, site of data collection, and contributing authors. Therefore, our review contained 19 unique study samples.

**Table 1:** Study characteristics

Author (year)	Geographical location and level of care	Study design	# of TBI participants (*n*) Mean age (SD)	Males (*n*, %)	Seniors > 65 (*n*, %)	Quality of life measure	Quality Assessment Score/18
Azouvi et al. (2016) [24]	Paris, France, Hospital and Community	Prospective cohort	85 42 (20)	69, 81.18%	9, 10.59%	QOLIBRI	12
Brown et al. (2004) [25]	New York, USA Community	Cross sectional	200 38.2 (16.0)	147, 55%	24 (range 60–99) 5.29%	Flanagan QOL Scale	13
Cnossen et al. (2017) [26]	Netherlands (NH); China (CH) Hospital	Prospective cohort (NH); Retrospective Cohort (CH)	NH: 447 46 (27-58) CH: 173 35 (24-50)	NH: 286, 64% CH: 116, 67%	NH: 63, 14.1% CH: 15, 8.7%	SF-36	13
Grieve et al.(2016) [27]	UK Hospital (Critical Care)	Cohort	3512 One mean NR	2687, 76.51%	86, 2.45% (70 +)	EQ-5D	11
Haller et al. (2016) [28]	Switzerland Hospital	Prospective cohort	351 Older group 74 (70–80)	257, 73.22%	97, 27.64%	SF-12	14
Horner et al. (2005) [29]	South Carolina, USA Community	Cross sectional	1606 NR	983, 61.20%	292, 18.2%	SF-36	16
Horner et al. (2008) [30]	South Carolina, USA Community	Cross sectional	1560 NR	953, 61.09%	287, 18.40%	SF-36	17
Kristman et al. (2016) [31]	Thunder Bay and Kingston, Canada Hospital (ED)	Prospective cohort	46 76.2 (7.4)	19, 41.30%	46, 100%	SF-12	17
Lin et al. (2016) [32]	Taipei, Taiwan Hospital and Community	Cohort (subset of survey data)	333 75.8 (8.4)	169, 50.8%	333 (range 60–99), 100%	QOLIBRI and WHO-QOL BREF	7
Mar et al. (2011) [33]	Basque Country and Navarre, Spain Hospital	Cross sectional	68 NR	37, 54%	48,70.6%	SF-36 and EQ-5D	14
Matuseviciene et al. (2016) [22]	Sweden Hospital (ED)	Randomized Control Trial	173 Single mean NR	78, 45.09%	14, 8.09%	SF-36	19* total possible score of 28
McCarthy et al. (2006) [34]	South Carolina, USA Hospital	Retrospective cohort	7612(weighted) 43.2 (20.0)	4865, 63.9%^a^	2272, 29.85%^a^ (range 55–75 +)	SF-36	15
Muehlan et al. (2016) [35]	Germany Community	Cross sectional	795 NR	NR	27, 3.40%	QOLIBRI-OS	10
Pickelsmier et al. (2006) [36]	South Carolina, USA Community	Prospective cohort	2118 NR	1284, 60.6%	500, 23.61%	SF-36	17
Selassie et al. (2009) [37]	South Carolina, USA Community	Cross sectional	2118 NR	1284, 60.6%	500, 23.61%	SF-36 and SIP	12
Stambrook et al. (1993) [38]	Manitoba, Canada Hospital	Cohort	106 Single mean NR	106, 100%	12 (11.3%)	Sickness Impact Profile (SIP)	11
Thompson et al. (2012) [39]	14 states, USA Community	Prospective cohort	414 NR	246, 59.3%	309, 74.64%	SF-36	16
Toman et al. (2017) [40]	Birmingham, UK Community	Cross sectional	124 NR	95, 76.61%	17, 13.71%	QOLIBRI	11
Toyinbo et al. (2016) [41]	Florida, USA Community	Cross sectional	485 35.0 (10.6)	454, 93.61%	9, 1.86%	NeuroQOL	8
Weber et al. (2015) [42]	Brazil Community	Prospective cohort	50 NR	44, 88%	5 (range 66-85), 10%	WHO-QOL BREF	9

#### Older adult study participants

Only two studies [31, 32] exclusively examined older adults (≥ 65 years). In two studies, three-quarters of the study sample consisted of older adults [33, 39]. In contrast, six studies reported fewer than of 10% of the study sample to be older adults [22, 24, 27, 35, 41, 42]. For ten studies in the review, 11–30% of the sample included older adults [25, 26, 28–30, 34, 36–38, 40]. Among 17 studies, less than half of the participants were female [24–30, 32–42], whereas two studies consisted of 55% females [22] and in one study 60% [31] were female participants.

#### Identification of Qol measures

Nine different Qol measures were identified among the 20 studies in the review, see Table 2. Seven main tools and two of the seven tools were abbreviated tools (SF-36 and SF-12 and the QOLIBRI with QOLIBRI-OS). Slightly more than half (11/20) of all studies reported using the Short Form Health Survey (9/11 used the SF-36 [22, 26, 29, 30, 33, 34, 36, 37, 39], while 2/11 used the short version SF-12[28, 31]). The Quality of Life after Brain Injury (QOLIBRI) measure was implemented in four studies; [24, 32, 35, 40], one of which used the 6 item QOLIBRI-OS [35]. The QOLIBRI-OS correlates well to the full QOLIBRI (*r* = 0.87) [43]. The World Health Organization Quality of Life BREF (WHO-Qol BREF) [32, 42] and the EuroQol-5D (EQ-5D) [27, 33] were each implemented two studies. The NeuoQol [41], Sickness Impact Profile (SIP) [38], and Flanagan Quality of Life Scale (FQolS) [25] were used in three studies. Three studies implemented two measures of Qol. The combinations of Qol measures included: QOLIBRI and WHO-QoL BREF [32], SF-36 and EQ-5D [33], and SF-36 and SIP[37].

**Table 2:** Qol instruments

QoL measure G-General or S-Specific	Number of dimensions (# of items)	Dimension categories	Psychometric properties	Feasibility in older adult population with TBI
SF-36 (G) SF-12 (G)	8 + 2 (36) 2 subscales (12)	Physical functioning, role-physical, bodily pain, general health, vitality, social functioning, role-emotional, mental health Abbreviated version of SF-36	Validity: + content validity in TBI population(g) Reliability: + inter-item correlation in TBI population [45] Validity + [46] Reliability: + [46]	More sensitive to capture difficulties of older adults with mild TBI as compared to those with moderate or severe TBI. Effects of depression may not be captured. Validated for TBI populations but mainly in studies with younger populations[45] International use: English and multiple other languages available [44] Cost: Manual and licensing fees [44] Administration: self, interviewer [44] Estimated completion time: < 10 minutes [44] Response options: binary and Likert options [44]
WHO-QoL BREF (G)	4 domains (26)	Physical, psychological, social, environment	Validity: + correlation with psychological well-being, social relationships, and physical capacity domains [47] Telephone mode has not been validated Reliability: + test–retest reliability in people with TBI, + test–retest with environment, social relationships, and physical and psychological domains [47]	Assessment in context of an individual’s culture, value system, personal goals, standards, and concerns. Used to measure recovery in the first year after TBI so has demonstrated to be sensitive to changes associated with rehabilitation including social support and depression [47]. Questions on employment may be seen as a sensitive question by some older adults who are retired High level of missing values for Q21 could be reflective of the sensitive nature of the question (How satisfied are you you’re your sex life)? [47] This measure is unique as it includes environment as a domain. This is important to the older adult as transportation, surroundings, the natural environment and can be environmental barriers that impact older adults with TBI International use: English and multiple other languages available [44] Cost: free [44] Administration: self, interviewer [44] Estimated completion time: < 10 minutes [44] Response options: Likert [44]
Quality of Life after Brain Injury (QOLIBRI) and Quality of Life after Brain Injury Overall Scale (S) QOLIBRI-OS (S)	6 domains (37) 6 domains (6)	Cognition, self, daily life and autonomy, social relationships, emotions, physical problems Abbreviated version of QOLIBRI	Validity :+ criteria validity, validated in older adults (> 74 years) [43, 48, 50] Reliability: + internal consistency + test–retest [43, 50] Abbreviated version has 1 question for each domain, may reduce validity and reliability [48]	First Qol tool specifically developed for person with TBI. Involves Qol and function measures and is seen as a more sensitive measure for clinical trials. Missing questions about whether the older person with TBI is bothered by seizures, legal issues, ability to drive, stigma, and sleeping problems [50] International use: English and multiple other languages available [49] Cost: free [49] Administration: self [49] Estimated completion time: < 10 minutes [49] Response options: Likert [49]
EuroQoL (EQ-5D) (G)	5 domains (15)	Mobility, self-care, usual activities, pain/discomfort, anxiety/depression	Validity: + construct validity [51, 52] Reliability: + test–retest in TBI population [51, 53]	Standard measure of health for clinical and economic appraisal. Vertical visual analogue scale reflects patient’s own judgment of their Qol, which is easy to complete and interpret. Standard data sets available for comparison by country but not by older adult age [52] International use: English and multiple other languages available [44] Cost: Licensing fee [44] Administration: self-reported, observer/proxy/telephone versions available [44] Estimated completion time: < 10 min [44] Response options: check boxes, visual analogue scales [44]
Sickness Impact Profile (SIP) (G)	12 (136)	Behavior, life participation, mental health ,social relationships :sleep and rest, emotional behavior, body care and movement, home management, mobility, social interaction, ambulation, alertness behavior, communication, work, recreation and pastimes, eating	Validity: + construct in TBI population [55] Reliability: + inter-rater reliability in TBI population [56, 57]	Normative data available for TBI ; however, mean age of study population was young (32 years, SD 13.1) [57] SIP has been used largely in those with moderate to severe TBI. [55] International use: English version only [54] Cost: Free [54] Administration: self, interview [54] Estimated completion time: 20–30 minutes [54] Response options: yes/no, check boxes [54]
The Flanagan Quality of Life Scale (QOLS) (G)	6 domains (16)	Physical and material well-being, relationships with other people, social community and civic activities, personal development and fulfillment, recreation, independence	Validity : − [59] Reliability: + internal consistency, + test–retest [59]	Developed in the 1970s for use with chronic illness groups. Developed with senior citizens in mind. This measures in largely cognitive based and fails to tap into the emotional domain [59] International use: English and multiple other languages available [58] Cost: Free; must contact copyright owner to use [58] Administration: self [58] Estimated completion time: < 10 minutes [58] Response options: 7-point Likert scale [58]
NeuroQoL	16 domains (564)	Physical, emotional, cognitive and social patient function	Validity : + in studies of neurological populations and pediatric TBI population and + content validity in military populations with TBI [4, 48, 61, 63] Reliability: + in studies of neurological populations and pediatric TBI population + internal consistency in military population with TBI [48, 60]	Measure can be used for adults and children; however, this may not take into consideration the unique challenges of an older adult. NeuroQol considered at item bank in which the assessment is individually tailored based on responses to previous items. Limited use if measures across groups are not the same so difficult to do a comparison across neurological patient groups [61] International use: English and multiple other languages available [62] Cost: Free [62] Administration: self, interviewer [62] Estimated completion time: < 10 minutes [62] Response options: Likert scale [62]

“+” = sufficient, “−” = insufficient, “?” = indeterminate as per COSMIN

### TBI severity and Qol measure

**Fig. 2:**
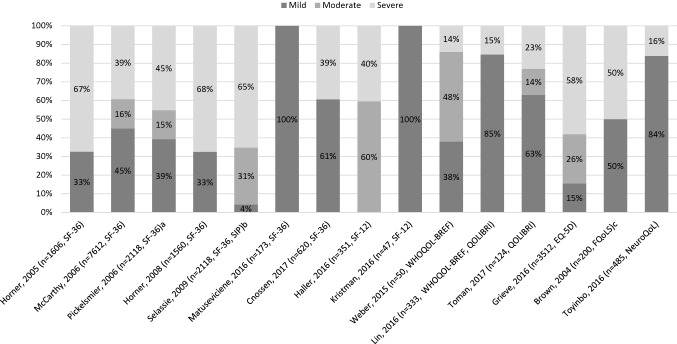
Percentage of participants by TBI severity. Stambrook et al. [38], Mar et al. [33], Thompson et al. [39], Azouvi et al. [24] and Muehlan et al. [35], did not report traumatic brain injury severity. a = study reported a sample size of 2118 people; reported severity scores for 2098 people. b = study reported a sample size of 2118 people; reported severity scores for 1947 people. c = study reported a sample size of 454 people; reported severity scores for 200 people.

Studies reporting TBI severity are illustrated in Fig. 2. Six studies included participants with all types of TBI severity (mild, moderate, severe) [27, 34, 36, 37, 40, 42]. The measures of Qol across these six studies varied. One study used the EQ-5D [27], two studies the SF-36,[34, 36] while another used SF-36 in combination with the SIP[37], one study used the QOLIBRI [40], and one the WHO-QoL BREF [42]. Of all 20 studies reviewed 14 reported some participants with mild TBI and among these studies about half (8/14) used the SF tools, SF-36 [22, 26, 28, 29, 34–37] and SF-12 [31], while the remaining 6/14 studies used FQolS [25], EQ-5D [27], WHO-Qol BREF and QOLIBRI together [32], QOLIBRI [40], NeuroQol [41], and the WHO-Qol BREF[42]. This review included a total of 7393 mild TBI participants, 3357 moderate TBI participants, and 10,114 severe TBI participants. The Qol measure dimensions including categories, total number of items measured, and the type of measure (general of specific) are presented in Table 2.

### Qol tool administration

Qol measures were administered by four different methods (phone, on-site interview, self-report, and surface mail). A total of seven different time points post injury (PI) were identified for Qol administration. Times included 0–30 days PI, 1 + to 3 months PI, 3 + to 6 months PI, 6 + to 12 months PI, 1 + to 3 years PI, 3 + to 5 years, and over 5 years PI). Four studies administered Qol measures across two time points [28, 31, 32, 39] and undertook comparison of Qol in the study analysis over time.

### Qualities of instruments and studies

Psychometric properties, using the COSMIN guideline, and feasibility of the Qol tool usage to older adults with TBI are presented in Table 2. We selected 22 articles to review the psychometric properties (reliability, validity, and feasibility in the older adult population with TBI) for the nine Qol measures we identified in this systematic review (see Table 2). Noted here are the references specifically used to develop Table 2, listed by each Qol measure with orientation to the specific article reviewed (and in this order) for validity, reliability, and feasibility: SF-36 and SF-12: Table 2 references [44–46]; WHO-Qol BREF: Table 2 references [44, 47], QOLIBRI, QOLIBRI-OS; Table 2 references [43, 48–50]; EuroQol: Table 2 references [44, 51–53]; SIP: Table 2 references [54–57]; Flanagan QOLS: Table 2 references [58, 59]; NeuroQol: Table 2 references [46, 60–63]. Construct or criteria validity was identified in the literature for six tools—SF-12 and 36, WHO-QoL BREF, QOLIBRI-OS, EQ-5D, SIP, NeuroQol, while reliability through test–retest methods and/or internal consistency was identified in the literature for six tools—WHO-Qol BREF, QOLIBRI, EQ-5D, SIP, Flanagan Quality of Life Scale, and NeuroQoL. Four of the seven tools were free to use, two required a licensing fee, and one of the tools is free to use after acquiring copyright permission. Each of the tools attempted to capture a range of physical, emotional, social/community, and spiritual health dimensions. Only the QOLIBRI is specific for brain injuries and captures the social relationships, emotions, physical problems, and self-domains of health. However, a prior study suggested that the QOLIBRI has missing questions regarding potential occurrences of seizures, legal issues, driving abilities, stigma, and sleeping problems. The SF-36, SF-12, and SIP have been validated for use within the TBI population and asks questions mainly regarding physical, social, and emotional/mental health. However, these tools have only been validated for and used mainly among younger TBI populations. All the Qol tools were self-reported and use Likert scales. Completion of each tool is estimated at less than ten minutes with the exception of SIP.

The Downs and Black with revision scores [5, 18, 20] are reported in Table 1 with detailed item specific scores in Table 3. The average score was 12.8/18 with a range of 7–17 (not including the randomized clinical trial by Matuseviciene [22]). Common methodological limitations across studies included missing data, limited or insufficient data on validity, and study power not addressed.

**Table 3:** Quality appraisal using Downs and Black [18] with revisions Baernholdt et al. [11], McHugh [23]

	1	2	3	5	6	7	9	10	11	12	16	17	18	20	25	26	27	Total
Azouvi et al. [24]	1	1	1	1	1	1	0	0	0	1	1	1	1	1	0	1	0	12
Brown et al. [25]	1	1	1	2	1	1	0	1	0	1	1	0	1	1	1	0	0	13
Cnossen et al. [26]	1	1	1	0	1	1	1	1	1	1	1	0	1	1	0	1	0	13
Grieve et al. [27]	1	1	1	1	1	1	0	0	1	0	1	1	1	1	0	0	0	11
Haller et al. [28]	1	1	1	1	1	1	1	1	0	1	1	1	1	1	0	0	1	14
Horner et al. [29]	1	1	1	2	1	1	1	1	0	1	1	1	1	1	0	1	1	16
Horner et al. [30]	1	1	1	2	1	1	1	0	1	1	1	1	1	1	1	1	1	17
Kristman et al. [31]	1	1	1	2	1	1	1	1	1	1	1	1	1	1	0	1	1	17
Lin et al. [32]	0	1	1	0	0	0	0	1	0	1	1	0	1	1	0	0	0	7
Mar et al. [33]	1	1	1	2	1	1	1	0	1	1	1	0	1	1	1	0	0	14
McCarthy et al. [34]	1	1	1	2	1	1	1	0	0	1	1	0	1	1	1	1	1	15
Muehlan et al. [35]	1	1	1	0	1	0	0	1	1	0	1	0	1	1	0	0	1	10
Pickelsimer et al. [36]	1	1	1	2	1	1	1	0	1	1	1	1	1	1	1	1	1	17
Selassie et al. [37]	1	1	1	1	1	1	0	1	1	0	1	1	1	1	0	0	0	12
Stambrook et al. [38]	1	1	1	1	1	1	1	0	0	0	1	0	1	1	1	0	0	11
Thompson et al. [39]	1	1	1	2	1	1	0	1	1	1	1	0	1	1	1	1	1	16
Toman et al. [40]	1	1	1	0	1	1	0	1	1	0	1	0	1	1	1	0	0	11
Toyinbo et al. [41]	1	1	1	0	1	0	0	0	0	1	1	0	1	1	0	0	0	8
Weber et al. [42]	1	1	1	0	1	1	0	1	0	0	1	0	1	1	0	0	0	9

	1	2	3	4	5	6	7	8	9	10	11	12	13	14	15	16	17	18	19	20	21	22	23	24	25	26	27	Total
Matuseviciene et al. [22]^a^	1	1	1	1	0	1	1	0	1	1	0	0	0	0	1	1	1	1	1	1	1	1	1	0	0	1	1	19

0 = no or unable to determine, 1 = partially, 2 = yes for item 5 only. In the Downs and Black—revised, 17 categories were used to identify the quality of each study, and they were as follows: (1) Hypothesis described. (2) Main outcomes described. (3) Patient characteristics described. (5) Principle confounders in each group described. (6) Main findings described. (7) Random variability of main outcomes. (9) Patients lost to f/u described. (10) Probability values. (11) Subjects representative of population. (12) Subjects representative of population they are recruited. (16) Data dredging. (17) Consistency in follow-up timing. (18) Appropriate statistics. (20) Main outcome measures accurate. (25) Adequate adjustment for confounding. (26) Patients lost to follow-up accounted for. (27) Sufficient power calculation reported

^a^Study was assessed using all of the items provided by Downs and Black. PROSPERO [16]

## Discussion

Older adults with TBI were not well represented in the literature when Qol is measured. Knowledge on the longer term impact of TBI on an individuals’ Qol as they age is important information for all age groups. Extrapolating study findings to older adults from study samples weighted largely towards younger adults is inadequate, as older adult-specific findings are diluted. Older adult females were largely underrepresented in the studies we reviewed. Sex was often reported by total number of participants only and not by age, thereby making it difficult to determine the sex ratio of older adults. The lack of studies with older adult female participants is of particular importance as older females may have very different Qol needs as compared to older adult males [64]. Moreover, most studies in the review were conducted in major cities of higher income countries. Therefore, little is known about Qol for those older adults living in remote and lower income countries, who may experience limited access to health care resources and face other barriers that impact their Qol.

The systematic review identified nine Qol measurements (7 measures, with 2/7 included abbreviated tools) used in studies that included older adults with TBI. Historically, Qol measures have fallen into two main categories: generic and disease/injury-specific tools [65]. Generic Qol measures are often used in health services research and population comparisons where the interest is in health status change across different diagnostic groups. In this review, seven of the nine Qol tools were generic measures (SF-36 and SF-12, WHO-Qol BREF, EQ-5D, SIP, FQolS, NeuroQol). The Short Form tools (SF-36, SF-12) have roots in the 1970s and have since been well documented for use in rehabilitation and many types of general diseases and injuries [66]. The EQ-5D tool has the added benefit of use in economic appraisal. It consists of 5 dimensions, is short and easy to complete, and has strong psychometric properties. It has been validated with a Canadian population and is recommended as a common data element for TBI by the National Institute of Neurological Disorders and Stroke [14]. Understanding the economic costs associated with Qol can be important given the strong financial impact of TBI on the health care system, particularly among older adults. Older adults with TBI have a rising number of emergency department visits, hospitalizations, and long recovery periods that require medical supervision [2, 13]. The WHO-Qol BREF is recommended in the data repository for the Ontario Brain Institute [67]. The WHO-Qol BREF was the only measure that included the assessment of factors in the physical environment. The NeuroQol was developed to assess domains of physical, mental, and social functioning for adults with a variety of neurological conditions and is also included in the NIH Toolbox measures recommended for use in research, clinical, and educational settings [14]. The SIP and the FQolS have not been reported in the literature for the past decade with use in TBI patients. The domains of the Qol generic measures identified included a broad range of concepts: physical and emotional health, self-care, pain, sleep and rest, activities of daily living, sexual functioning, and environment.

In contrast to generic tools are disease/injury-specific measures of Qol that are used by clinicians in practice to assess clinical changes within patients [65]. The disease/injury-specific measure of Qol is generally responsive to clinical changes over time [65]. The QOLIBRI and abbreviated version QOLIBRI-OS were the only TBI-specific Qol instruments and were implemented in four studies [24, 32, 35, 40]. QOLIBRI is recommended for the general adult population of TBI by the National Institute of Neurological Disorders and Stroke [13]. It has the potential to identify specific consequences of a TBI injury and it can also detect the effects of interventions.

The importance of a common Qol measure for older adults with TBI is predicated on their unique characteristics, which includes changes in pathophysiology, higher incidence of general brain deterioration, co-morbid medical problems, reduced psychosocial and financial support, decreased motivation, and lowered expectations for recovery [14]. A suitable Qol measure must also take into consideration pre-injury disability. Dimensions that were not addressed in the either the generic or injury-specific measures reported but are worthy of future consideration in a common measure for the older adult post TBI include nutrition [68], medication use and quality of sleep [64], social cohesion and aspects of their built environment that could affect safety [69], vision [70], and physical activity [71] and community engagement. Understanding the relationship between the older adults’ individual needs and their physical environment may require both subjective and objective measures. For example, people with TBI regularly encounter physical barriers in the community such as steep slopes, stairs, curbs, and narrow pathways that can limit their performance and engagement in their community and subsequently impact their quality of life. One study in our review included both an injury-specific and generic measure of Qol [32] which may serve as a best practice approach for clinicians and researchers.

TBI is a highly heterogeneous injury by cause, severity, pathology, age, sex, clinical course, and patient outcomes. Based on the diversity of TBI outcomes by severity of injury, one could speculate that there may be need for TBI severity-specific Qol tools (i.e., tools specific to mild, moderate, or severe TBI). However, in the review no clear pattern emerged on Qol measurement tool use by TBI severity. We calculated the sum of all study participants in the review and found twice as many total study participants with moderate or severe TBI as compared to mild TBI. This is a concern as 80% of all TBIs are mild TBI and many are unreported, missed, or not assessed [16]. Challenges faced by researchers in the identification and recruitment of older adults with mild TBI may be the reason for this underrepresentation of older study subjects. Innovative methods are required to identify older adult patients with mild TBI for research studies, as they may not seek health care services, but may be suffering from a mild TBI in silence.

## Limitations

Measuring Qol after TBI poses several significant methodological challenges. Unlike organ based diseases, where blood tests can help guide diagnosis and treatment, there are currently no rapid, definitive diagnostic test for TBI. Adding to this challenge is the fact that TBI is a group of injuries that are highly diverse by cause, severity, age, sex, symptoms, and premorbid history. The studies in this review represent a very broad spectrum of TBI care and recovery that can limit comparison and critical appraisal. In addition, there may be bias in the study sample over time as those who survive a TBI and return to the community may represent a select group of older adults with relatively fewer health problems, fewer cognitive, physical and emotional challenges, and better psychosocial support. The systematic review only included published studies in the English language. Although rigorous selection criteria were employed to ensure methodological quality and consistency, we were unable to confirm eight studies claiming the sample age range that included older adults actually meet the study criteria (> 5 adults ≥ 65 years of age).

Our purpose was to identify the Qol measures used in older adult with TBI. The literature we selected to review on validity and reliability of the nine measures using the COSMIN criteria was not a comprehensive. However, it is worth considering that there may be limited evidence on the methodological strength of these instruments in the TBI population of older adults. No content mapping of the measures was included in the review to evaluate if domains of measures cover areas of importance to individuals with TBI. In addition, our review has limited information regarding the ability of the Qol measures to detect change and interpretability of measures. Further investigation of the Qol measures using the COSMIN criteria is warranted.

## Conclusions

We identified nine Qol measures that have been used in studies that included older adults with TBI. Findings based on the comparison of reliability and construct validity of the measures reported in this review suggest that no single instrument is superior to all others, for our study population. Future research in this field should include the enrollment of larger study samples of older adults. Without these future efforts, the ability to detect an optimal Qol measure will be hindered. As long as researchers and clinicians continue to use different tools to measure Qol, differences in outcomes could be a result of differences in measurement rather than understanding the unique rehabilitation needs of this population.

## Appendix

Database: Ovid MEDLINE(R) In-Process and Other Non-Indexed Citations and Ovid MEDLINE(R) < 1946 to Present > Search Strategy:

**Table:**

1	exp brain injuries/[including smaller terms: brain concussion, post concussive syndrome, brain hemorrhage, traumatic, brain injury chronic, diffuse axonal injury, pneumocephalus] (49186)
2	concuss$.ti,ab. (3817)
3	post?concussion.ti,ab. (361)
4	traumatic brain injur$.ti,ab. (18503)
5	(brain injury adj2 traum$).ti,ab. (17989)
6	tbi.ti,ab. [tbi in title or abstract only] (13096)
7	exp craniocerebral trauma/(120500)
8	head injuries, closed/(2621)
9	(head injur$ adj3 closed).ti,ab. (2226)
10	or/1-9 (128583)
11	limit 10 to (“all aged (65 and over)” or “aged (80 and over)”) (19051)
12	exp aged/[includes MeSH terms ‘aged 80 and over’ and ‘frail elderly’] (2356669)
13	advanced age$.ti,ab. (11066)
14	advancing year$.ti,ab. (154)
15	agedness.ti,ab. (4)
16	ag?ing.ti,ab. (140485)
17	elder$.ti,ab. (181430)
18	retire$.ti,ab. (13409)
19	pension$.ti,ab. (3002)
20	(old$ adj2 (age$ or patient$ or m?n or wom?n or male$ or female? or person$ or people$ or population)).ti,ab. (473217)
21	senior.ti,ab. (20069)
22	or/11-21 (2809465)
23	10 and 22 (23542)
24	exp “Quality of Life”/(119676)
25	(quality adj2 life$).mp. [mp = title, abstract, original title, name of substance word, subject heading word, keyword heading word, protocol supplementary concept, rare disease supplementary concept, unique identifier] (199290)
26	exp adaptation, psychological/(102082)
27	attitude/(39275)
28	questionnaires/(304753)
29	exp Health Status Indicators/(201237)
30	health status inventor$.mp. (27)
31	Positive–negative evaluation.mp. (4)
32	pne.mp. (383)
33	HRQOL.ti,ab. (7595)
34	Rand 36.mp. (529)
35	SF12.mp. (160)
36	sf-36.ti,ab. (13380)
37	qol.ti,ab. (19573)
38	or/24-37 (747435)
39	23 and 38 (1378)
40	remove duplicates from 39 (1314)
41	limit 40 to english language (1215)
	(1614 up-date)
